# Diverse and Complementary Effects of Ghrelin and Obestatin

**DOI:** 10.3390/biom12040517

**Published:** 2022-03-29

**Authors:** Daniel Villarreal, Geetali Pradhan, Yu Zhou, Bingzhong Xue, Yuxiang Sun

**Affiliations:** 1 Department of Nutrition, Texas A & M University, College Station, TX 77843, USA; dvillar77@gmail.com; 2 USDA/ARS Children’s Nutrition Research Center, Department of Pediatrics, Baylor College of Medicine, Houston, TX 77030, USA; gpradhan@purdue.edu; 3 Interdepartmental Program in Translational Biology and Molecular Medicine, Baylor College of Medicine, Houston, TX 77030, USA; 4 Department of Physiology and Pathophysiology, School of Basic Medical Sciences, Qingdao University, Qingdao 266071, China; yuzhou@qdu.edu.cn; 5 Department of Biology, Georgia State University, Atlanta, GA 30303, USA; bxue@gsu.edu

**Keywords:** ghrelin, obestatin, GHS-R, GPR39, gastric peptides

## Abstract

Ghrelin and obestatin are two “sibling proteins” encoded by the same preproghrelin gene but possess an array of diverse and complex functions. While there are ample literature documenting ghrelin’s functions, the roles of obestatin are less clear and controversial. Ghrelin and obestatin have been perceived to be antagonistic initially; however, recent studies challenge this dogma. While they have opposing effects in some systems, they function synergistically in other systems, with many functions remaining debatable. In this review, we discuss their functional relationship under three “C” categories, namely complex, complementary, and contradictory. Their functions in food intake, weight regulation, hydration, gastrointestinal motility, inflammation, and insulin secretion are complex. Their functions in pancreatic beta cells, cardiovascular, muscle, neuroprotection, cancer, and digestive system are complementary. Their functions in white adipose tissue, thermogenesis, and sleep regulation are contradictory. Overall, this review accumulates the multifaceted functions of ghrelin and obestatin under both physiological and pathological conditions, with the intent of contributing to a better understanding of these two important gut hormones.

## 1. Introduction

The process of deriving multiple protein isoforms from a single gene is referred to as alternative splicing. Alternative splicing is a major mechanism contributing to the protein variants of post-transcriptional and post-translational modification [1]. Many protein isoforms are encoded by the same genes but exhibit distinctive properties. For example, the BCL2L1 gene encodes two isoforms: the long isoform inhibits apoptosis, while the short isoform promotes it. Genome sequencing has revealed that the number of protein-coding genes does not match the complexity of an organism [2]. Isoforms derived from the same gene are known to interact with different signaling pathways, leading to distinctive functions. The extent to which these “sibling proteins” are present in organisms may serve as an explanation for the inherent complexity between mammals and simple organisms.

Gastrointestinal peptides ghrelin and obestatin are encoded by the same gene, ghrelin. Ghrelin regulates a wide range of biological functions, such as appetite control, energy expenditure, gastrointestinal (GI) motility, gastric acid secretion, insulin secretion, etc. [3,4,5,6,7]. The circulating levels of ghrelin and obestatin exhibit a differential profile during fasting; ghrelin levels increase while obestatin levels decrease [8]. Obestatin affects the gastrointestinal system, pancreas, adipose tissues, and cardiovascular system [9]. The key functions of ghrelin and obestatin are known to be antagonistic: ghrelin enhances appetite and increases food intake; in contrast, obestatin suppresses appetite and reduces food intake. However, while this contradictory nature was once regarded as a common phenomenon, recent evidence reveals that the functions of ghrelin and obestatin are much more complex. This review recapitulates the gene structure, expression, and functional diversity of these two “sibling proteins” under both physiological and pathological conditions.

## 2. Gene Structure, Post Translational Processing, Expression, and Putative Receptors

**Figure 1:**
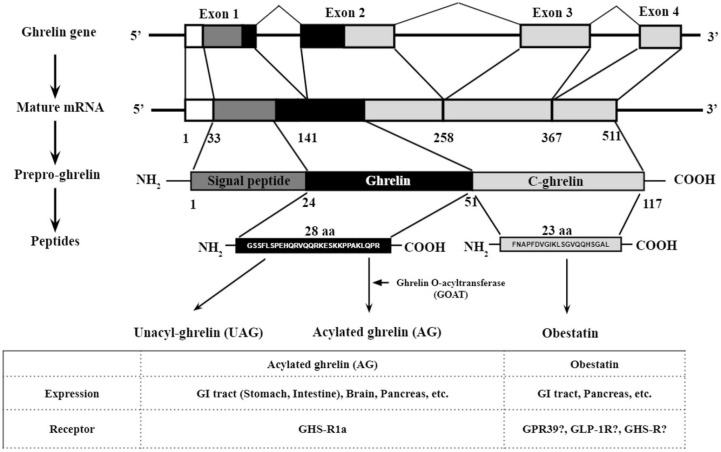
Post-translational processing of preproghrelin to unacyl ghrelin, ghrelin, and obestatin. The figure was modified from Delporte [10].

Ghrelin and obestatin are derived from the same ghrelin gene following post-translational cleavage of the 117 amino acid pre-proghrelin peptide [10]. Mature pre-proghrelin is then post-translationally modified by signal peptidase, prohormone convertase 1/3 (PC 1/3) and carboxypeptidase-B like enzyme into 28 amino acid unacyl ghrelin (UAG) and 23 amino acid obestatin; the unacyl ghrelin peptide is further post-translationally modified into acyl ghrelin, also known as ghrelin (Figure 1). Acylation of ghrelin is potentiated by ghrelin O-acyltransferase (GOAT) [11]. Growth Hormone Secretagogue receptor (GHS-R), a G-protein coupled receptor (GPCR), is the biologically relevant receptor for ghrelin [12,13,14]. The acylation of ghrelin is essential for its binding to GHS-R [15]. Traditionally, acyl ghrelin has been considered the biologically active isoform, whereas UAG has been considered the biologically “inactive” isoform [16]. Subsequent studies have revealed that UAG also has biological functions, although the receptor of UAG is still unknown [17]. Obestatin is produced by the post-translational modification of the same pre-proghrelin peptide following post-translational amination of the C-terminal [3], which is essential for the stable conformation of obestatin [9,18]. Currently, the receptors of obestatin and the enzymes involved in obestatin processing are still unclear.

Ghrelin is predominantly secreted by the GI system (e.g., stomach, duodenum, and intestine), and lower levels of ghrelin are detected in many other tissues, such as the brain, pituitary, pancreatic islets, kidney, lung, heart, and ovaries [19,20]. Similarly, obestatin is also widely expressed in a variety of tissues, such as the gastrointestinal tract, skeletal muscle, adipose tissue, lung, liver, pancreatic islets, mammary glands, and testis [19,20,21,22,23,24]. Both ghrelin and obestatin exist in plasma, saliva, breast milk, and semen [25]. In contrast to ghrelin, the expression of the ghrelin receptor GHS-R is much more restricted. High levels of GHS-R expression can be detected primarily in the pituitary and brain but to a much lesser extent in peripheral tissues [15,26]. Interestingly, GHS-R expression in some tissues appears to be age-dependent. We have reported that GHS-R expression is very low in the brown adipose tissue (BAT) and white adipose tissue (WAT) of young mice, but it is increased in these tissues of older mice [8,14,26].

Although GHS-R is the recognized receptor for ghrelin, the receptor for obestatin has been an ongoing debate. G-protein coupled receptor 39 (GPR39), glucagon-like peptide-1 receptor (GLP-1R), and GHS-R have been proposed to function as receptors for obestatin [3,27,28]. However, the in vivo functional relevance of these putative receptors has not been determined. Zhang et al. reported that obestatin is the cognate ligand for GPR39, based on the observation that obestatin induces c-fos expression in wild-type mice but not in GPR39-null mice [3]. Other reports show that obestatin inhibits lipogenesis in adipocytes [29] and influences HL-1 cardiomyocyte functions via GPR39 [30]. However, some studies dispute that obestatin is the endogenous ligand for GPR39, due to a lack of confirmed signal transduction and specific binding on GPR39-expressing cells [27,31]. Interestingly, Zn^2+^ has been shown to activate GPR39 in various tissues, including pancreas, bones, neurons, salivary glands, colon, skin, heart, and prostate cancer cells [32,33,34,35,36,37,38]. A recent review described the multiple roles of GPR39 in brain homeostasis, for example, maintaining excitatory/inhibitory tone of neural circuit and vascular tone of microcirculation, regulating inflammatory tone, and functioning as a positive allosteric agonist [39]. In intestinal L- and K-cells, oral administration of Zn^2+^ induces activation of GPR39, which stimulates GIP secretion and leads to increased insulin secretion and glucose tolerance [40]. Even at a physiological concentration, Zn^2+^ released from injury sites or vesicles is sufficient to activate GPR39 [41,42]. Some recent studies have further suggested that Zn^2+^ at physiological conditions acts as a modulator of GPR39 [39]. There is evidence that Zn^2+^ binding to GPR39 results in receptor activation, subsequently, stimulating downstream signaling mediators, such as Gα_q_, Gα_s_, Gα_11/12_, and β-arrestin [38]. All the studies above are in support of Zn^2+^ as the endogenous ligand of GPR39. 

Others postulate that obestatin’s satiety and anorectic properties are potentiated via binding with incretin receptors, such as the glucagon-like peptide-1 receptor (GLP-1R). Similar to obestatin, incretins reduce GI motility, promote satiety, and reduce food intake [43,44]. GLP-1R has been reported to bind to obestatin in human beta cells to promote islet survival [27]. Obestatin upregulates GLP-1R expression and increases beta-cell survival, and the effect of obestatin on islet survival can be blocked by GLP-1R antagonists. However, studies utilizing INS-1 pancreatic beta cells and HEK293 kidney cells overexpressing GLP-1R failed to detect obestatin interaction [45], which questions whether GLP-1R is indeed the biologically relevant receptor for obestatin. We have reported that obestatin can function via GHS-R in pancreatic beta cells during hyperglycemic conditions because we observed that GHS-R ablation abolishes obestatin-induced insulin secretion [28]. Our finding suggests that obestatin’s stimulatory effect on insulin secretion is mediated by GHS-R; however, obestatin may not necessarily directly bind to GHS-R. It is possible that obestatin affects the downstream signaling of GHS-R or interacts with a heterodimer of GHS-R. Overall, to date there is still no consensus on the obestatin receptor.

## 3. Obestatin and Ghrelin: Complex, Contradictory, and Complementary

It has been generally accepted that the functions of obestatin and ghrelin are antagonistic. This theory was propagated following the discovery that obestatin opposes ghrelin’s effects on food intake [3]. While many studies support the opposing roles in some systems, other studies have revealed synergistic and complementary roles in other systems. This review discusses the functional complexity of ghrelin and obestatin by exploring the 3 “C”—the complex, complementary, and contradictory functions—and aims to enhance our understanding of the biology and applications of these important gut hormones.

### 3.1. Complex Functions

#### 3.1.1. Food Intake, Weight Regulation, GI Motility, and Hydration

The emergence of obestatin as the antagonistic ‘sibling’ of the ghrelin gene has inspired researchers to postulate its potential therapeutic uses in combating obesity-related diseases. The decision to name the peptide ‘obestatin’ was based on the proposed function of ‘obesity suppression’. However, further research on the antagonistic functions revealed a more complex picture. Obestatin was initially studied in relation to ghrelin’s well-known functions in food intake, weight regulation, and gastrointestinal motility [3]. While ghrelin is primarily known for its stimulatory effect on food intake, weight gain, and gastric emptying [12,46,47]; obestatin has been reported to inhibit food intake, reduce bodyweight, and suppress jejunal motility [3]. The suppressive effects on food intake and the reduction in weight gain were observed under both acute and chronic exposures to obestatin. Thus, while the antagonistic relationship is supported in some studies [48,49,50,51,52], other studies do not substantiate this conclusion [31,45,53,54,55,56,57,58].

A recent report showed that chronic administration of obestatin to high-fat diet-fed rats was able to prevent/protect the occurrence of non-alcoholic fatty liver disease (NAFLD) [59]. They observed improvements in hepatic insulin signaling, reduced hepatic lipid accumulation, increased circulating adiponectin, inhibited ghrelin acylation, and reduced food intake/body weight. It has also been reported that a combination of obestatin and CCK8, a satiety signal produced in response to dietary fat, elicits significant body weight reduction by 29%, while obestatin alone only reduces bodyweight by 13% [60]. A report demonstrated that the truncated version of obestatin is more effective in inhibiting food/water intake [61], which may explain the discrepancies in some studies. These confounding results may be due to several factors, such as the use of human or rat/mouse obestatin, preparation of peptide, site of administration (central/peripheral), the strain of species, time of day of administration, nutrition status (fed/fast), etc.

Interestingly, it has been suggested that any appetite-suppressing effect of obestatin is secondary to its ability to inhibit water intake due to the suppression of vasopressin [62,63]. It was initially reported that obestatin cannot cross the blood–brain barrier, and it is quickly degraded in circulation [64,65]. Obestatin administered via intracerebroventricular (ICV) delivery was found to influence the subfornical organ, a group of neurons responsible for fluid regulation. This suggests that brain-derived obestatin [66], not peripheral obestatin, plays a dominant role in the regulation of hydration in mice [63]. There are conflicting results on whether ghrelin influences water intake. The administration of ghrelin has been shown to inhibit water intake following an acute stimulus of peripheral hypertonic saline and central angiotensin II (AngII), but the effect disappears following a 24 h dehydration period [67]. In summary, the inability to consistently replicate the antagonistic functions of obestatin challenges the conclusion that obestatin exclusively functions as an antagonistic rival of ghrelin.

#### 3.1.2. Diverse Expression Profiles under Fed and Fasted Conditions

Another area of contention is the role of obestatin in the fed and fasted states. Obestatin’s role has been investigated to a lesser degree than that of ghrelin, as its primary functions under fed/fasted conditions remain controversial. Zhang et al. reported that, in response to a 48 h fast and refeeding in rats, obestatin concentrations stayed constant in both fasted and fed states [3]. Another study showed that ghrelin and obestatin are elevated in rats after 48 h of fasting, and are decreased by feeding [68]. Further, it was demonstrated that plasma obestatin in mice is significantly decreased, whereas plasma acyl ghrelin is increased following a 24 h fast [69]. We found that overnight fasting increases acyl ghrelin but not unacyl ghrelin in young and middle-aged mice, but not in old mice, while overnight fasting decreases obestatin in all ages [8]. Still, others have reported that a 24 h fast in mice results in a similar elevation of both ghrelin/obestatin, showing an inverse relationship with insulin/leptin [70]. These differences may depend on the species used, the duration of the fast, and the metabolic states of the animals tested.

Ghrelin signaling has been well studied in severe undernutrition and weight loss conditions, such as anorexia nervosa (AN) [71]. In humans, ghrelin levels were significantly elevated in AN patients [72,73,74,75]. In a study comparing AN patients with BMI-matched healthy subjects, it was shown that AN patients have doubleted fasting plasma ghrelin levels, suggesting that ghrelin might be important in the pathogenesis and development of AN [76]. A similar observation was also made in animal models of anorexia nervosa [77,78]. Further, in AN patients that regained weight, there was a 25% reduction in fasting plasma ghrelin [72], normalized ghrelin [79], or ghrelin levels even below control subjects [80]. Similar to ghrelin, obestatin concentrations are diminished following a glucose bolus in non-anorexic women but remain elevated in anorexic women [81,82] and in AN patients with restrictive food behavior (AN-R), while they decrease in AN patients who develop episodes of bulimia (AN-BP) [83]. All ghrelin-related peptides (acyl/unacyl ghrelin and obestatin) and glucose levels are elevated in anorexic individuals, while plasma insulin levels remain low [84]. Other studies have reported similar elevations of obestatin in response to anorexia [85]. Interestingly, in subjects with higher body mass indexes (BMIs), both peptides are significantly reduced under fasting conditions, suggesting their involvement in the regulation of energy balance [84]. A high-carbohydrate breakfast also induces a decrease in plasma ghrelin and obestatin in AN patients. Ghrelin/obestatin ration is also lower in AN patients compared to controls in the postprandial period [82]. However, Behçet’s disease patients exhibit high levels of circulating obestatin during fasting and reduced prevalence of obesity, suggesting a crucial role of obestatin in energy regulation [86]. Additionally, obestatin expression in human gastric mucosa is significantly diminished in abdominal obesity with distended waists and normal BMI, correlating with increased insulin resistance and elevated cholesterol and triglycerides [87]. This phenomenon is reflected in obese children, where circulating obestatin is lower compared to controls, and is associated with increased fasting insulin, LDL and leptin [88]. The levels of ghrelin and obestatin in a mixed group of obese males and females suggest that while both peptides are reduced in circulation, the ratio of ghrelin/obestatin is increased [89,90]. However, other studies have found that in obese women, obestatin levels are elevated, ghrelin levels are reduced, and the ghrelin/obestatin ratio is decreased [90]. This observed reduction in ghrelin/obestatin ratio with the obese cohorts was further substantiated by a meta-analysis study, which illustrated the relationship between the ghrelin-related peptides in normal weight and obese subjects following an overnight fast [91]. Nine studies with over 500 participants found that obestatin in normal subjects was, on average, 64.19 pg/mL higher than obese subjects. Twenty-one studies with over 1000 participants found that the total ghrelin of normal weight subjects was, on average, 145.53 pg/mL higher than obese subjects. Furthermore, in 5 studies including over 200 participants, the ratio of ghrelin/obestatin is 2.49 pg/mL higher than in normal-weight subjects. These results show that the obestatin, acyl-ghrelin, and total ghrelin levels are significantly higher in normal-weight subjects than obese subjects, suggesting an important role for ghrelin-related peptides in maintaining a healthy bodyweight. In the current literature, there is no consensus regarding the relationship of ghrelin/obestatin under either fed or fasted states, but there is agreement on the reduction of the ghrelin/obestatin ratio during obesity and elevation of obestatin in anorexic individuals.

#### 3.1.3. Blood Pressure, Cardiovascular Disease, and Inflammation

The literature surrounding obestatin’s role in cardiovascular health and inflammation is currently in its nascent stages. Fasting plasma obestatin levels are negatively correlated with systolic blood pressure in humans [92], but obestatin levels are increased in hypertensive rats [93]. However, hypertensive rats exposed to a range of obestatin concentrations experienced no difference in mean arterial pressure, heart period, or baroreflex sensitivity [94]. Moreover, obestatin is thought to regulate blood pressure, as its concentration is positively correlated with systemic blood pressure in women with normal pregnancy and pregnancy-induced hypertension [95]. Both ghrelin and ghrelin/obestatin ratios are significantly lower in patients with mild-to-moderate untreated hypertension compared to normal controls [96]. As mentioned earlier, obestatin inhibits dehydration-induced vasopressin secretion, consequently influencing electrolyte homeostasis and blood volume [62,63]. The administration of exogenous obestatin to obese and lean subjects has been shown to result in improved forearm blood flow in both groups. This vasodilatory effect is interpreted as being due to obestatin’s ability to enhance nitric oxide activity [97]. Ghrelin’s levels in circulation have been shown to be inversely corelated to blood pressure [98], and exogenous administration of ghrelin reduces blood pressure in normotensive animals [99], healthy people [100], animals and people experiencing heart failure [101,102], and animals with hypertension [103]. Additionally, acyl-ghrelin administration in healthy males is found to improve heart rate, blood pressure, body surface temperature, and respiratory rate via stimulation of parasympathetic tone. In summation, while ghrelin is known to be inversely related to blood pressure, the physiological relevance of obestatin to blood pressure regulation is complicated by differences in species, health status and sex difference [98]. While ghrelin lowers blood pressure in animals [103] and humans [104], exogenous obestatin improves vasodilation in lean and obese human subjects [97] but has no effect on hypertensive rats [94]. Thus, while there are promising revelations concerning obestatin’s effect on blood pressure, the literature on that has yet to reach a consensus.

The role of obestatin in cardiovascular disease (CVD) and inflammation is another area that deserves further investigation, as it may serve as an important biomarker for the severity and progression of chronic inflammatory diseases. Obestatin has been reported to elicit cardioprotective properties, and speculation has arisen over possible clinical use of the peptide as a biomarker for cardiac-related morbidities [30,63,105,106,107]. Indeed, obestatin levels correlate to the onset of diabetic nephropathy (hyperglycemia-induced damage of the kidneys) [108]. A sign of emerging nephropathy is the inability of the kidneys to properly filter the blood, which is reflected by the increased appearance of microalbuminuria (albumin in the urine) or proteinuria (protein in the urine). Microalbuminuria is a strong independent indicator of cardiovascular risk in those with or without T2D [109]. In T2D patients with microalbuminuria, there are significantly higher concentrations of serum obestatin than in T2D patients without microalbuminuria. This suggests that increased levels of obestatin in T2D may be tied to the progression of diabetic nephropathy, as well as cardiovascular risk. However, other studies have reported low obestatin associated with obesity, insulin resistance, and visceral adiposity [87,88,91]. An explanation for this phenomenon could be that, as T2D becomes more severe, obestatin levels increase as a compensatory mechanism for beta-cell preservation [27,110,111]. Beta-cell compensation has been reported in cases of pancreatitis [112]. Thus, rising obestatin levels may serve as an important biomarker for the emergence of diabetic nephropathy and a risk indicator for adverse cardiovascular events.

Interestingly, obestatin also plays a significant role in the later stages of chronic kidney disease. It is noteworthy that diabetic nephropathy precedes chronic kidney disease, which is associated with a decreased glomerular filtration rate. For each ng/mL increase in serum obestatin, there is an associated decrease in mortality among maintained hemodialysis patients (MHD) [106]. Incidence of death from cardiovascular issues is correlated with increased levels of obestatin, and this association is even more pronounced in patients >71 years old. The interaction between low obestatin and high IL-6 is associated with an increased risk of mortality causes (synergy index [SI] = 5.14), as well as cardiovascular-related deaths ([SI] = 4.81). The conclusion of this study suggests that obestatin is a prominent biomarker for predicting the survivability of MHD patients. The correlation of simultaneous elevation of obestatin and kidney disease progression suggests that obestatin may have a compensatory role in attenuating the severity of kidney disease.

There are also reports showing that the combination of low obestatin and high TNF-α significantly increases the mortality of MHD patients [84]. Furthermore, in vitro work suggests that, in the absence of TNF-α, obestatin may be atherogenic due to its ability to increase oxidized-LDL uptake in macrophages [113]. This results in foam cell formation, which is a hallmark of atherogenesis. Conversely, obestatin suppresses vascular cell adhesion molecule-1 (VCAM-1) expression in the presence of TNF-α, effectively eliciting an anti-inflammatory effect. This result suggests that obestatin-induced modulation of atherogenesis is dependent on inflammatory status. This reinforces the notion that obestatin acts as a compensatory mechanism in response to inflammatory conditions. These studies describe an interesting phenomenon: high levels of obestatin in T2D may imply the emergence of diabetic nephropathy [108], as an indicator of increased cardiovascular risk, while high levels of obestatin in patients undergoing chronic hemodialysis may reflect enhanced survivability and CVD-related mortality [106]. Intriguingly, it has been observed that obestatin increases the accumulation of pro-inflammatory macrophages under normal conditions [113] but delays the onset of atherogenesis under elevated TNF-α condition and reduces the mortality risk of hemodialysis patients [106,107]. These contextual functions are extremely complex, warranting further investigation to determine whether obestatin can be used as a biomarker to assess the progression of cardio-renal disease.

In comparison, ghrelin’s role in CVD and related morbidities is better documented. The beneficial effects of ghrelin regarding the cardiovascular system include enhancing myocardial contractility, reducing mean arterial pressure/vasodilatation, attenuating heart failure, improving ventricular remodeling, and protecting myocardium from ischemia and reperfusion injury [114,115,116]. Increased angiogenesis following ghrelin administration in rodent models via activation of the pro-survival Akt—vascular endothelial growth factor—Bcl-2 signaling cascade has also been reported [117]. Ghrelin treatment reduces inflammatory responses, apoptosis, and oxidative stress induced by cardiopulmonary bypass (CPB) and preserves the cardiac pumping function via GHS-R–Akt signaling [118]. Ghrelin treatment in a rat model of myocardial infarction (MI) results in increased vascular endothelial growth factor (VEGF) expression and enhanced angiogenesis [119]. Additionally, systemic ghrelin administration increases VEGF expression but decreases nitric oxide (NO) in diet-induced obese mice [120]. The literature surrounding ghrelin’s role in CVD is predominantly in support of the attenuation of symptoms and damage, while obestatin’s role is still largely not well defined.

#### 3.1.4. Insulin Secretion

The roles of ghrelin and obestatin in energy homeostasis are well established and recognized as orexigenic or anorexic peptides, respectively; studies of these two peptides on insulin secretion are less established. Obestatin has been shown to modulate glucose-stimulated insulin secretion [27,28,121,122]. We have shown that obestatin increases insulin secretion using in vitro, ex vivo, and in vivo models and revealed that obestatin’s stimulatory effect on insulin secretion is GHS-R dependent [28]. Others reported that obestatin promotes insulin release from islets with low or no glucose [27]. Interestingly, Egido et al. reported that obestatin potentiates a dual effect on insulin secretion [121]. They reported that insulin is stimulated under low glucose concentrations, while it is inhibited under high glucose. Ample evidence shows that ghrelin inhibits insulin secretion in animals [123,124,125], and the blockade of ghrelin enhances insulin secretion and ameliorates the development of diet-induced glucose intolerance [126]. We reported that ghrelin deletion increases glucose-induced insulin secretion and improves glycemic control in leptin-deficient *ob/ob* mice by reducing uncoupling protein 2 (UCP2) in pancreatic islets [127]. In summation, the role of obestatin in glucose-stimulated insulin secretion is still contested and requires further investigation, while ghrelin’s insulinostatic properties are better established.

### 3.2. Complementary Functions

#### 3.2.1. Pancreatic Beta Cell Protection

The convergence of the synergistic effects of ghrelin and obestatin is best exemplified by their complementary roles in preserving the function of pancreatic beta cells. Both ghrelin and obestatin are expressed in the fetal and adult endocrine pancreas, suggesting that they may regulate the development and function of pancreatic islets [128]. Granata et al. reported that treatment with obestatin enhanced the expression of genes associated with insulin biosynthesis, beta cell survival/differentiation, and upregulation of GLP-1R [27]. Obestatin has been shown to improve beta cell function and survival via activation of the cAMP response element binding (CREB) protein and increased expression of pancreatic and duodenal homeobox 1 (Pdx1), which is important in glucose sensing, β-cell differentiation, and insulin production. Obestatin also increases the expression of glucokinase and insulin receptor substrate-2 (IRS-2), which has been implicated in compensatory beta-cell hyperplasia in response to high-fat diet-induced insulin resistance [27,129,130]. This highlights the potential autocrine/paracrine role of obestatin. Furthermore, obestatin treatment in the culture of human islets has led to activation of CREB, a primary regulator of β-cell survival and glucose homeostasis, as well as enhanced expression and phosphorylation of IRS-2 mRNA [131]. Obestatin is also implicated in driving pancreatic development and regeneration, suggested by the activation of fibroblast growth factor receptors, notch receptors, and neurogenin-3, resulting in increased insulin gene expression and the formation of pancreatic islet-like clusters [110]. Obestatin is reported to be secreted by human pancreatic islets and pancreatic beta cell lines, which provide protective effects from starvation, inflammatory insult, and apoptosis [27,111]. Furthermore, it is suggested that the anti-apoptotic property of obestatin is due to increased islet vascularization [111]. In rats in response to acute pancreatitis, obestatin increases the blood supply to the pancreas, reduces inflammation, decreases digestive enzyme activity, and improves pancreatic regeneration [132,133]. Increased circulating obestatin is observed in patients experiencing acute pancreatitis, suggesting a compensatory mechanism [112].

Additionally, both ghrelin and obestatin have been shown to protect rats from streptozotocin (STZ)-induced beta cell death, evident of increased islet area, islet number, beta-cell mass, upregulated insulin and PDX1 mRNA, and improved glucose metabolism [134]. Moreover, ghrelin is reported to potentiate a trophic effect, protecting β-cells from damage in an experimental model of T1DM [7,135]. T1DM rats treated with ghrelin have higher survival rates (68% compared to 11%) and exhibit improved glucose tolerance and insulin secretion [136]. It is suggested that the effect is due to reduced lymphocyte infiltration, enhanced beta cell proliferation, and increased neogenesis-driven Pdx1 expression. This evidence suggests that ghrelin may have therapeutic potential for type 1 diabetes. Obese and diabetic mice administered exogenous ghrelin before meals have been shown to significantly increase GLP-1, which leads to improved glucose tolerance and insulin secretion [137]. In summary, ghrelin and obestatin potentiate similar protective effects on islet function through their own respective pathways; further investigation of their roles in the endocrine pancreas is warranted as ghrelin and obestatin may provide novel therapeutic options for diabetes.

#### 3.2.2. Muscle Function

Muscle function and protection are impacted by both ghrelin and obestatin. In response to muscle injury, the ghrelin gene is upregulated, suggesting that both obestatin and ghrelin are integral to muscle maintenance and repair [23,138]. Obestatin directly stimulates muscle regeneration via the increased activity of satellite cells in several models of muscle injury [139,140]. Obestatin exerts a significant effect on muscle regeneration via an autocrine mechanism to control the GPR39-mediated myogenic differentiation program [23]. The expression of myogenic genes in rats is upregulated following obestatin infusion, supporting the role of obestatin in muscle regeneration. Overexpression of both obestatin and GPR39 in skeletal muscle improves regeneration after acute muscle injury, exhibiting upregulation of myogenic factors (Pax7, myogenin, MyoD) and increasing myofiber size [139]. Additional findings further revealed that intramuscular injections of obestatin improve muscle regeneration via upregulation of VEGF/VEGFR2, microvascularization, and inhibition of myostatin [139]. In skeletal muscle, obestatin not only promotes regeneration but also plays a role in fiber type determination; obestatin-treated muscle shows upregulation of Mef2 [141]. Obestatin promotes the activity of protein kinase D (PKD) and Ca^2+^/calmodulin-dependent protein kinase (CAMK) to promote the phosphorylation of class II HDACs. This signaling activation results in the translocation of class II HDAC from the nucleus to cytosol, subsequently activating Mef2 and Mef2-dependent genes, as well as transcriptional factors regulating type 1 slow-twitch fibers. Furthermore, obestatin enhances the expression of PGC1-α, a master regulator of mitochondrial biogenesis and oxidative metabolism, which is reflected by the increase in Cytochrome C, uncoupling protein 3 (UCP3), and Carnitine palmitoyltransferase 1 (CPT1) [141]. Interestingly, while AMPK regulates mitochondrial biogenesis and fiber type determination, the administration of obestatin does not affect AMPK; instead, it potentiates the Akt/mTOR signaling pathway to promote myotube growth [141]. Obestatin attenuates muscle injury via regeneration of the satellite cell pool [140], enhances the expression of myogenic genes [139,142], increases micro-vascularization (via upregulation of VEGF/VEGFR), enlarges myofiber size [139], and affects fiber type determination [141]. These findings collectively suggest that obestatin plays an important role in muscle mass maintenance and fiber type determination, which may be a promising therapeutic strategy for muscle wasting diseases.

Similarly, ghrelin possesses its own muscular-protective effects via different pathways. The role of ghrelin in regulating muscle function is based on its prevention of atrophy and its anti-inflammatory effects. Ghrelin indirectly increases muscle mass by stimulating GH/Insulin-like growth factor-1 axis, enhancing food intake, and promoting positive energy balance in cachexic mice [143]. Ghrelin attenuates cachexia in multiple pathological conditions, such as heart failure, chronic obstructive pulmonary disease, Huntington’s disease, and thermal injury [102,143,144,145,146,147]. In C2C12 myoblasts, ghrelin administration promotes the differentiation and fusion of myocytes [148]. Remarkably, the muscle wasting condition of fasting- and denervation-induced muscle atrophy is prevented by ghrelin treatment via activation of Akt/mTOR signaling [149]. We showed that ghrelin protects against fasting-induced muscle atrophy in aging mice [150]. Others reported that muscle injury in *ghrelin*^−/−^ mice exhibits significantly impaired muscle regeneration due to diminished satellite cell pool and self-renewal [151]. Additionally, attenuated muscle regeneration was postulated due to a lack of anti-inflammatory properties potentiated by ghrelin-related peptides via GHSR-independent mechanisms [151]. The regenerative properties of ghrelin are also observed in models of Huntington’s disease (HD). Daily injection of ghrelin preserves weight, reverses the expression of catabolic genes, improves skeletal morphology, and mitigates behavior deficits associated with HD pathogenesis in R2/6 mice [147]. In the case of muscle function, ghrelin attenuates HD-induced skeletal muscle loss, maintains muscle mass during fasting via Akt/mTOR signaling [149], and promotes muscle regeneration through its anti-inflammatory properties [151]. In summary, both ghrelin and obestatin are essential in the protection, regeneration, and maintenance of skeletal muscle mass, and the effects are mediated via both common and distinctive signaling pathways.

#### 3.2.3. Neuronal Function/Injury and Parkinson’s Disease

As previously discussed, both peptides elicit similar beneficial effects in certain tissues but do so via different mechanisms; this is evident in the regulation of neuronal function, as well as their response to injury. For example, centrally administered obestatin is reported to improve memory retention in rats via interaction with the hippocampus and amygdala [51]. Ghrelin treatment improves memory retention, synaptic plasticity [152], memory task performance, and long-term potentiation, as well as increases dendritic spine density in the hippocampus and hypothalamus [153]. It has been suggested that obestatin improves cerebral circulation via enhanced nitric oxide synthase expression in microvascular endothelial cells [154], which subsequently leads to improved vasodilation of central arteries [155]. This vasodilatory function of obestatin also exists in the systemic circulation system [156]. Interestingly, while ghrelin improves endothelial cell nitric oxide production in systemic arteries, it does not function in the same way in the endothelial cells of central arteries [155]. In addition, obestatin has been proposed as an anti-seizure therapy, as it was found to improve the neuronal survival and memory function of normal and epileptic mice via attenuation of lipid peroxidation and oxygen radicals [157]. Similarly, ghrelin is implicated in the attenuation of epileptic seizures by interacting with neuropeptide Y (NPY) or GABA neurons [158].

Interestingly, both peptides have been reported to ameliorate the progression and symptoms of Parkinson’s disease (PD) via improvements in dopamine neurons. PD pathogenesis relies on the degeneration of dopamine neurons located primarily within the substantia nigra [159]. Both peptides seem to produce similar neuroprotective effects in dopaminergic cell lines. Obestatin treatment exhibits neuroprotective properties in dopaminergic MES23.5 cells and in neurotoxin-induced injury [15]. The protective effects of obestatin are not due to the inhibition of apoptosis but due to an enhancement of proliferation, which differs from the reported anti-apoptotic properties of ghrelin in MPP-treated MES23.5 cells [160]. The PD pathology of the degeneration of dopamine neurons within retinal cells results in impaired vision. Obestatin treatment induced anti-apoptotic effect in the retinal ganglion cell line (RGC-5) exposed to H_2_O_2_ oxidative stress, resulting in increased expression of Bcl-2 [161]. Obestatin potentiates the protective effect via the activation of TrkB-Akt/ERK1/2 signaling; the administration of exendin (GLP-1R antagonist) partially inhibits this protective effect. These results suggest that obestatin has therapeutic potential for PD. Similarly, ghrelin treatment also elicits a protective effect in RGC-5 cells exposed to rotenone [162]. Ghrelin treatment resulted in the increased expression of Bcl-2, attenuation of apoptosis, and improvement of mitochondrial function by modulating GHS-R/Akt/mTOR signaling. Collectively, these findings suggest that both peptides influence memory retention, vasodilation, and neuronal survival in several models of injury. These reports substantiate the notion that obestatin and ghrelin act complementarily to potentiate similar benefits via diverse mechanisms.

#### 3.2.4. Cancer

Currently, the understanding of obestatin in cancer is limited. Most of the research has focused on the impact of obestatin on gastric cancers. Administration of obestatin to human gastric cancer KATO-III cells results in enhanced cell proliferation via activation of ERK1/2 signaling pathway [163]. The unidentified obestatin receptor is thought to interact with PI3K, then PI3K signaling subsequently activates a novel PKCε, responsible for MAPK activation. Álvarez et al. reported that obestatin-induced proliferation in KATO-III and gastric adenocarcinoma cells (AGS) is medicated by the activation of GPR39-facilitated recruitment and activation of Src [164]. Src promotes the activation of metalloproteinases (MMPs) to stimulate EGFR, which subsequently activates PI3K. The activation of PI3K turns on Akt, which inhibits mTORC1, followed by downstream activation of p70S6K1 and promotion of cell proliferation. More recently, the obestatin/GPR39 system has been implicated in the pathogenesis of gastric adenocarcinomas, suggesting that obestatin regulates human gastric adenocarcinoma cells via GPR39 [165]. The immunostaining of Ki-67, a marker of cellular proliferation, suggests that obestatin may interact with GPR39 to increase AGS proliferation, exhibiting a protective effect. GPR39 activation of the β-arrestin/MMP/EGFR/Akt/mTOR pathway may be partially responsible for the morphological and functional changes seen in AGS cells [165]. Taken together, these studies provide critical insight into obestatin in gastric cancers, and further investigation of the obestatin/GPR39 system in cancer is warranted.

Multiple cancer studies suggest a role for ghrelin and GHS-R in various tumors, modulating proliferation, apoptosis, and metastasis [166,167,168,169]. In pituitary tumors, ghrelin mRNA is expressed in gonadotropin and GH-producing adenomas, non-functional adenomas, and prolactinomas. GHS-R expression is significantly elevated in GH-producing adenomas [170]. In patients with advanced pancreatic cancer, aging and anorexia are associated with reduced circulating active ghrelin [171]. Ghrelin and GHS-R are also relevant in the context of prostate cancer pathology. Human prostate carcinomas and benign neoplasms express the mRNA of ghrelin and GHS-R [172]. However, in normal prostatic tissue, the expression of ghrelin mRNA is undetectable, suggesting that ghrelin is involved in the pathogenesis of prostate cancer. Ghrelin and its synthetic mimetics modulate the proliferation of various prostatic cancer cell lines. That has been controversial, as some studies suggest a proliferative effect on cancer cell growth [173,174], while others observe stimulatory property at physiological dose and inhibitory property at pharmacological doses [172]. It is interesting to note that the expression of GOAT, the enzyme responsible for ghrelin acetylation, is significantly upregulated in human prostate cancer cells [175].

Zhu et al. reported that, in human non-small cell lung cancer A549 cells, ghrelin administration stimulates proliferation [176]. This effect is accompanied by the activation of GHS-R and its associated signaling of PI3K/Akt/mTOR/P70S6K and ERK. Ghrelin’s proliferative property is attenuated in the presence of PI3K, mTOR, and ERK inhibitors, while GHSR siRNA attenuates the phosphorylation of PI3K, Akt, ERK, mTOR, and P70S6K. Similarly, GHS-R antagonists attenuate ghrelin-induced human colon cancer cell proliferation via inhibition of the Ras/PI3K/Akt/mTOR pathway [177]. Other studies suggest that ghrelin’s proliferative property is not exclusively dependent on GHS-R activation [178,179,180]. In both human intestinal cells and colon cancer cell lines (Caco-2), the administration of acyl and unacyl ghrelin stimulates proliferation [181]. Furthermore, this effect is attenuated by a non-selective GHS-R antagonist but not GHS-R siRNA, suggesting that it is a GHS-R independent mechanism.

#### 3.2.5. Digestive System

Ghrelin and obestatin have been shown to have protective effects on various organs of the digestive system. In the oral cavity, ghrelin is secreted by the parotid and submandibular salivary glands [182], taste buds of the tongue, and gingival epithelium [183,184]. Ghrelin inhibits the production and release of proinflammatory IL-8 in epithelial cells [184]. Simultaneously, proinflammatory IL-1β can increase the expression of *Ghsr* mRNA in periodontal cells [185]. These findings suggest that ghrelin is protective against inflammation. Oral mucositis severely affects quality of life and is associated with severe oral pain, dysphagia, odynophagia, and dehydration [186]. It has been shown that administration of ghrelin helps in the healing of oral ulcers, and this healing effect is associated with reduced mucosal IL-1β and improved mucosal blood flow and cell vitality [187]. In the esophagus, Thomas et al. reported that a higher concentration of ghrelin was associated with an increased risk of Barrett’s esophagus compared to control subjects [188]. Continuous infusion of ghrelin after esophagectomy reduces the duration of systemic inflammatory response syndrome (SIRS) by lowering C-reactive protein and IL-6 [189]. Further, a reduced level of plasma ghrelin could indicate longer SIRS duration after esophagectomy [190].

In the stomach, ghrelin has been reported to have protective effects in various models of gastric ulcers. Exogenous administration of ghrelin inhibits ethanol-induced gastric ulcers potentially by lowering the development of gastric lesions, increasing blood flow, and reducing TNF-β expression [191,192]. Pretreatment with ghrelin also protects against gastric ulcers induced by water immersion, restrain stress, concentrated hydrochloric acid, and alendronate [193]. Additionally, ghrelin stimulates the healing of gastric ulcers induced by various agents [194,195]. In the small intestine, ghrelin protects against damage induced by ischemia/reperfusion probably by reducing the proinflammatory cytokines and neutrophil infiltration, as well increased intestinal blood flow [196,197]. It has also been shown that ghrelin protects the small intestine after whole-body irradiation [198] and improves intestinal barrier functions after cerebral hemorrhage [199]. Besides having protective effects, ghrelin also aids in the healing of duodenal ulcers [195,200] and enhances intestinal adaptation after resection surgery [201].

The protective effect of ghrelin has also been observed in the liver; it has been demonstrated in various animal models of liver injury, including acetaminophen, bile duct ligation, and liver injury-induced ischemia/reperfusion [193]. Low ghrelin is associated with an increased risk of gallstone disease [202], but high ghrelin appears to reduce the risk of nonalcoholic fatty liver disease (NAFLD) [203,204,205]. Further, administration of ghrelin has been shown to be preventative and therapeutic for NAFLD [206]. In the pancreas, pretreatment with ghrelin prevents the development of acute pancreatitis induced by cerulein and pancreatic ischemia [207,208,209]. Furthermore, treatment with ghrelin after the onset of acute pancreatitis accelerates the recovery process [210,211]. Clinical reports show that in patients with pancreatitis, the level of ghrelin increases gradually and is the highest at discharge, suggesting that endogenous ghrelin plays a role in the recovery process of pancreatitis [186].

In the large intestine, *ghrelin* and *Ghsr* mRNA are higher in patients with inflammatory bowel disease (IBD) than in healthy controls [212,213]. Treatment with ghrelin significantly reduces the effects of colitis and accelerates healing in various experimental models of colitis [213,214,215,216]. In a clinical study, patients with ulcerative colitis exhibited upregulation of *ghrelin* and *TNF-α* mRNA compared to healthy subjects [213]. Also, we have recently shown that global deletion of GHS-R in mouse model exacerbates dextran sulfate sodium (DSS)- induced experimental colitis [217]. The protective effect of ghrelin on colitis could be due to reduced activation of nuclear factor kappa B (NF_k_B) which prevents breakdown of intestinal barrier function and inhibition of cell apoptosis [218,219]. However, some studies in *ghrelin*^−/−^ mice, *Ghsr^−/−^* mice and mice with knockdown of ghrelin-O-acyltransferase (GOAT) have suggested that ghrelin enhances colitis by promoting release of proinflammatory cytokines [220,221,222].

Various studies have demonstrated that exogenous ghrelin inhibits the expression and release of proinflammatory cytokines, including IL-1β, IL-6, IL-8, and TNF-α, in the oral cavity, esophagus, stomach, liver, pancreas, and colon [193]. Ghrelin can inhibit the translocation of NF_k_B into the nucleus and reduce MAPK signaling, lowering the production of proinflammatory cytokines [209,219,223,224,225]. Besides reducing the inflammatory response, the healing effects of ghrelin in ulcers have been attributed to increased mucosal blood flow, improved cell vitality and proliferation, and reduced oxidative stress in mucosa [186].

Similar to the protective and healing effects of ghrelin on the digestive system, obestatin has also been associated with protection and healing of gastric ulcers induced by acetic acid [226,227,228], and protection against trinitrobenzene sulfonic acid-induced colitis [229]. Additionally, administration of obestatin reduces the lesions of colon mucosa in acetic acid-induced acute colitis [227]. It has been reported that in peptic ulcer disease, the concentration of obestatin changes with the progression of infection [230]. A higher concentration of obestatin is associated with the severity of pancreatitis in humans [112]. Obestatin protects against acute pancreatitis induced by cerulein [133] and pancreatic ischemia after reperfusion [231], exhibiting a healing effect [232,233]. In the liver, obestatin protects against ischemia-induced hepatic injury and NAFLD [59,234]. Khaleel et al. reported that administration of obestatin protects against NAFLD, reduces accumulation of lipids in the liver, and develops hepatomegaly, hyperlipidemia, and insulin resistance [59]. Various studies have suggested that the protective and healing effects of obestatin are caused by the improvement of blood flow, improved cell vitality and proliferation, and reduced expression of IL-1β and TNF-α [226,229].

### 3.3. Contradictory Functions

Up to this point, most functions ascribed to ghrelin and obestatin are either complementary or synergistic; the following section covers the generally established contradictory functions.

#### 3.3.1. Lipid Metabolism

Obestatin regulates glucose metabolism in adipose tissue. It has been reported that obestatin translocates GLUT4 to the plasma membrane via the activation of sirtuin1 and Akt [235]. In adipocytes, obestatin activates key insulin signaling regulators of Akt, glucometabolic regulators of glycogen synthase kinase-3β (GSK3β), and master metabolic regulators of mechanistic targets of rapamycin (mTOR) [23]. Obestatin inhibits basal- and insulin-stimulated lipogenesis in adipocytes via GPR39–1a [29]. Furthermore, reduced glycerol release was observed in GPR39-deficient adipocytes cultured with obestatin, suggesting that the lipolytic effect of obestatin may be GPR39-dependent. Conversely, continuous ghrelin administration is known to promote adiposity by increasing food intake and decreasing fat utilization [236]. Ghrelin administration leads to increased adiposity accompanied by the stimulation of lipid storage enzymes, while simultaneously inhibiting the rate-limiting step of fatty acid oxidation [237]. Ghrelin has been shown to increase adipogenesis, stimulate pre-adipocyte differentiation, and antagonize lipolysis [238]. Generally, ghrelin promotes fat deposition and suppresses lipolysis, while obestatin inhibits lipogenesis and promotes lipolysis.

#### 3.3.2. Thermogenesis

The thermogenic activity of brown adipose tissue (BAT) is positively correlated with energy expenditure, and dysregulation of thermogenesis in BAT is linked to obesity in humans [239]. BAT, a key organ of non-shivering thermogenesis, plays an important role in energy expenditure. BAT contains large amounts of mitochondria, where lipids are used to generate heat [240,241]. Evidence suggests that non-shivering thermogenesis plays a crucial role in boosting energy expenditure in rodents and human neonates [239,240,242,243]. Enhanced thermogenesis also improves glucose homeostasis and insulin sensitivity in mice [244] and humans [245]. Upon cold-stimulus, the sympathetic nervous system (SNS) is stimulated, which releases norepinephrine (NE) into BAT to activate the β3-adrenergic receptor (β3-AR). Subsequently, thermogenic regulator uncoupling protein 1 (UCP1) is recruited into mitochondria, which promotes lipolysis and heat production [240,241].

Ghrelin has been reported to suppress NE release in BAT [246,247], and ghrelin suppresses brown fat thermogenesis via regulation of UCP1 [246,247,248,249,250]. In humans, the inhibitory thermogenic effect of ghrelin was observed in a study of men undergoing non-shivering cold exposure [245]. In response to cold exposure, activation of BAT thermogenesis results in a significant decrease in leptin, gastric inhibitory peptide (GIP), glucagon, and ghrelin. We reported that ghrelin and obestatin have opposite effects on UCP1 expression in brown adipocytes in a dose-dependent manner [8]. The results suggest that ghrelin and obestatin may have opposite effects on thermogenesis, leading to opposing outcomes for energy expenditure. Intriguingly, we found that *Ghsr*-null and *ghrelin*-null mice have a deferential thermogenesis phenotype: while *Ghsr*-null mice have elevated brown fat thermogenesis, thermogenesis in *ghrelin*-null mice is not altered [251]. Both ghrelin and obestatin are absent in *ghrelin*-null mice, and the opposing thermogenic effects of ghrelin and obestatin likely neutralize each other. Indeed, we found that deletion of the *Ghsr* gene results in increased thermogenesis, leading to a lean and insulin-sensitive phenotype in aging [248]. In *Ghsr*-null mice, only ghrelin signaling is blocked, while obestatin is intact. We believe this explains why only *Ghsr*-null mice, not *ghrelin*-null mice, exhibit an enhanced thermogenic phenotype. However, little data are currently available regarding the direct effect of obestatin on thermogenesis, and much more research needs to be done.

#### 3.3.3. Sleep

Obestatin and ghrelin also have differential effects on sleep. An interest in the relationship between the onset of sleep and feeding-related hormones has developed due to the revelation that metabolic syndrome is associated with sleep disorders [252]. Ghrelin and obestatin are two such hormones that have opposing effects on sleep. It has been revealed that ghrelin and other appetite-stimulating peptides induce wakefulness, while obestatin and other satiety peptides have a sleep-promoting effect after central but not systemic administration [252,253,254,255]. The use of ghrelin gene knockout mice bolstered the role of ghrelin-related peptides’ importance in sleep [254]. While mice are fasted and exposed to sub-thermoneutral conditions, they experience hypothermic bouts associated with a reduction in sleep; however, when treated with obestatin, it delays the onset of hypothermia and improved sleep [254]. Moreover, central administration of obestatin via ICV injections promoted the onset of sleep during the dark period; this is reflected by increased non-rapid eye movement (NREM) sleep [252], which is linked to improvement of hippocampal-memory consolidation [255]. In summary, ghrelin promotes lipogenesis, inhibits thermogenesis, and induces wakefulness, while obestatin promotes lipolysis, increases thermogenesis, and promotes sleep.

## 4. Conclusions

**Figure 2:**
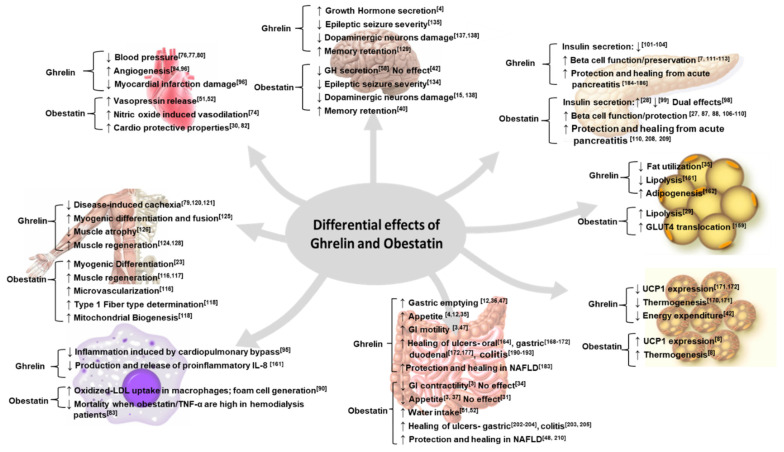
Summary of the differential effects of ghrelin and obestatin. ↑ denotes increase in function. ↓ denotes decrease in function [3,4,7,8,12,15,23,27,28,29,30,31,34,35,36,40,42,47,48,51,52,74,76,77,79,80,82,83,87,88,90,94,95,96,98,99,101,102,103,104,106,107,108,109,110,111,112,113,116,117,118,120,121,124,125,126,128,129,134,135,137,138,159,161,162,164,168,169,170,171,172,183,184,185,186,190,191,192,193,202,203,204,205,208,209,210].

The “sibling proteins” ghrelin and obestatin, encoded by the same ghrelin gene, have distinctive biological functions. The functional properties of these 2 peptides are very complex and can be either opposite, similar, or complementary depending on the tissue types and physiological/pathological states. Ghrelin and obestatin are involved in a wide range of physiological functions and become dysregulated under pathological conditions. In Figure 2, we summarize the functions of ghrelin and obestatin in various tissues under different physiological/pathological conditions. The commonly noted antagonistic functions are primarily evident in their effects on food intake and sleep: ghrelin increases food intake, while obestatin decreases food intake; ghrelin induces wakefulness, while obestatin promotes sleep. In this review, we have also discussed their regulation of thermogenesis and fat metabolism: ghrelin suppresses thermogenesis and promotes fat deposition, while obestatin increases thermogenesis and promotes lipolysis. We also discussed other differential functions: ghrelin potentiates anti-inflammatory effects and protects against inflammation, while obestatin’s effect on inflammation depends on the pathological states; ghrelin’s insulinostatic property of suppressing insulin secretion is well established, while obestatin’s effect on insulin is controversial. In addition, we also noted that they both display protective effects on pancreatic beta cells, muscle, neuronal health, and the digestive system, but elicit detrimental effects on the progression and metastasis of cancers. Thus, as our understanding of ghrelin and obestatin grows, the dogmatic view of ghrelin and obestatin as rivalry peptides has become less accurate. Their purported opposing properties are not ubiquitous in all tissues; while they have antagonistic effects in some tissues, they possess similar/synergistic effects in other tissues, and they activate distinctive signaling pathways. In Table 1, we summarize the signaling pathways utilized by ghrelin and obestatin in various tissues.

Taken together, we conclude that the biology of ghrelin and obestatin is complex; despite deriving from the same gene, they have both diverse and complementary effects, which can be opposite, similar, or synergistic depending on tissues/conditions. To advance their therapeutic applications, more in-depth studies are warranted to further define the functional diversity of these two sibling peptides. Specifically, it would be beneficial to determine the underpinning mechanisms promoting divergent functions and identify the differential signaling pathways used by these two proteins, thereby further advancing the understanding of the biological diversity associated with these two very important hormones.

## Figures and Tables

**Table 1:** Summary of signaling pathways of ghrelin and obestatin.

	Acylated Ghrelin (AG)	Obestatin
(1) Food intake	GH release via GHSR activation [12,13,14]	Down-regulation of NPY and NPY-R [37,42]
(2) Insulin signaling	AMPK signaling mediated G-protein subunit activation [101]	Regulation of pScr, Akt, ERK1/2 via GLP-1R binding [27]
(3) Thermogenesis	Suppression of NE release of BAT [170,171]	Up-regulation of UCP1 mRNA expression [8]
(4) Neuronal Function	Neuronal survival of epileptic mice via attenuation of lipid peroxidation [134]	Attenuation of epileptic seizure duration/onset via interaction with neuropeptide Y (NPY) or GABA neurons [135]
(5) Muscle function	Activation of GH/IGF-1 via mTOR/Akt signaling [120,126]	Regulation of myogenic differentiation through involvement of GPR39 [23]
(6) Cardiovascular function	Cardiac pumping function via GHSR and Akt signaling [95]	Activation of PI3K, PKCs and ERK1/2 pathway [82]

## Data Availability

Not applicable.
